# Community-associated Methicillin-resistant *Staphylococcus aureus*, Singapore

**DOI:** 10.3201/eid1102.040782

**Published:** 2005-02

**Authors:** Li-Yang Hsu, Anne Tristan, Tse-Hsien Koh, Michéle Bes, Jerome Etienne, Asok Kurup, Thuan-Tong Tan, Ban-Hock Tan

**Affiliations:** * Singapore General Hospital, Singapore;; † Faculté de Médecine Laënnec, Lyon, France

**Keywords:** letter, community-associated methicillin-resistant *Staphylococcus aureus*, multi-locus sequence typing, Panton-Valentine leukocidin

**To the Editor:** Community-associated methicillin-resistant *Staphylococcus aureus* (CA-MRSA) has been reported from almost every continent (*1**–**4*). Such strains are usually characterized by susceptibility to multiple non-β-lactam antimicrobial drugs, production of Panton-Valentine leukocidin (PVL), and presence of staphylococcal chromosome cassette *mec* (SCC*mec*) IVa, a novel smaller variant of the methicillin-resistance locus (*5*). The genetic backgrounds of CA-MRSA strains from different parts of the world are distinct and specific for each geographic region (*1**–**5*).

We conducted a study at our institution, a 1,600-bed adult acute-care, tertiary-level public hospital, to determine evidence and the clinical and molecular profile of CA-MRSA in Singapore. We reviewed the microbiology laboratory records at our institution for multidrug-susceptible MRSA strains isolated from January 1, 2001, to April 15, 2004. *S. aureus* was identified by colony morphologic features, coagulation of citrated rabbit plasma with EDTA (BBL Becton Dickinson and Co., Cockeysville, MD, USA), and production of clumping factor and protein A (BactiStaph, Remel, Lenexa, KS, USA). Methicillin resistance was determined by susceptibility testing and confirmed by latex agglutination for penicillin binding protein-2a (*6*). Multidrug-susceptible strains were defined by susceptibility to cotrimoxazole and gentamicin as determined by the Kirby-Bauer disk diffusion method following NCCLS guidelines (*7*).

The medical records of patients infected by these MRSA were reviewed, and strains were labeled community-associated if they had been isolated within 48 hours of hospitalization from patients who had not been in any hospital for >1 year. Community-associated strains were tested for PVL genes (*8*), and the SCC*mec* was typed by following a previously described method (*9*). Molecular typing was done by pulsed-field gel electrophoresis (PFGE) with restriction endonuclease *Sma*I and multilocus sequence typing (*10*). These strains were sent to the French Reference Center for Staphylococci, where genetic sequences encoding accessory gene regulator (agr) subtypes, enterotoxins, exfoliative toxins, toxic-shock syndrome toxin-1, hemolysins, and *LukE-LukD* leukotoxin were detected by polymerase chain reaction (PCR)–based methods. Comparisons with CA-MRSA strains worldwide in terms of toxin profile and PFGE patterns were also performed in France; the latter was achieved by using Taxotron software (Institut Pasteur, Paris, France) to digitize and analyze *Sma*I macrorestriction patterns.

Eight of 266 multidrug-susceptible strains fulfilled the criteria for community acquisition, but only 5 of these strains (corresponding to patients 1, 3, and 6–8) had been archived. The demographic and clinical data of the patients are shown in the Table. Most were young, healthy adults with cutaneous abscesses. Patient 1 had diabetes mellitus but had never been hospitalized; he was the only patient with severe bacteremic pneumonia. Patient 6 had early-stage endometrial cancer resected in 2000 but had not attended her follow-up appointments for >1 year before her hospitalization. Patient 8 had traveled to Taipei, Taiwan, for a month; the abscess developed 3 days after her return home. Travel history was not documented in the other patients' records. Patients 2–8 received β-lactam antimicrobial drugs in addition to surgical drainage of their abscesses and recovered without any complications.

**Table:** Demographic and clinical data of patients with community-associated methicillin-resistant *Staphylococcus aureus* (MRSA)

Patient	1	2	3	4	5	6	7	8
Date of MRSA isolation	Mar 2001	Nov 2002	Jan 2003	Feb 2003	Mar 2003	May 2003	Oct 2003	Apr 2004
Ethnicity	Indian	Filipino	Chinese	Chinese	Filipino	Chinese	Filipino	Chinese
Age	52	20	38	37	31	56	21	33
Sex	M	F	M	M	F	F	F	F
Coexisting conditions	Diabetes mellitus	–	–	–	–	Endometrial cancer	–	–
Infection type	Pneumonia, bacteremia	Hand abscess	Hand abscess	Hand abscess	Hand abscess	Hand abscess	Chin abscess	Abdominal wall abscess
Therapy*	IV vancomycin	I&D	I&D	I&D	I&D	I&D	I&D	I&D
Appropriate antimicrobial drug usage	Yes	No	No	No	No	No	No	No

*Therapy: IV, intravenous; –, not applicable; I&D, incision and drainage of abscess.

All 5 archived strains had different molecular and toxin profiles, and the only consistent feature was the presence of PVL genes. Isolates 3 and 7 possessed SCC*mec* IV. Isolates 1, 6, and 8 were *mec*A positive, but their SCC*mec* belonged to none of the 4 major structural types. Comparisons with published data on CA-MRSA strains showed that isolate 7 was identical to the European strain of CA-MRSA in terms of PFGE pattern, toxin profile, and sequence type (ST 80) (*2**,**5*). Isolate 3 had an identical PFGE pattern and sequence type (ST 30) compared to the Oceanian Southwest Pacific strain but differed slightly in toxin profile, as the *LukD-LukE* leukocidin genes were absent (*3**,**5*). Isolate 8 was similar to the Taiwanese strains: it was ST 59 and had non-typable SCC*mec* (*4*). It belonged to agr 1 and tested positive for enterotoxin *sek*, γ2-hemolysin, and β-hemolysin genes.

Isolate 6 had a PFGE pattern that may be distantly related to U.S. strains; the similar sequence type (ST 1) served to emphasize this, although the presence of nontypable SCC*mec* rather than SCC*mec* IV implied that methicillin resistance was acquired differently. It belonged to agr 3 and tested positive for *LukD-LukE* leukocidin, enterotoxins *seb* and *seh*, and γ2-hemolysin genes. Isolate 1 is unique to Singapore in that it had a novel sequence type (ST524: 7-6-1-5-71-5-6) and SCC*mec*. It belonged to agr 1 and tested positive for γ-hemolysin gene as well as for the enterotoxin gene cluster.

Widely diversified CA-MRSA strains exist in Singapore. The demographic profile and clinical symptoms of local patients infected with these strains were consistent with published literature (*2**–**4*). The lack of a pediatric unit at our institution prevented a more complete epidemiologic description.

In contrast to previous reports (*1**–**5*), our findings are unique in that most of our strains do not have a distinctive molecular profile and may be related to strains from different parts of the world. Epidemiologic and molecular data strongly suggest that isolate 8 was imported from Taiwan. Some of the other strains (especially isolates 3 and 7) may have been imported from other countries too, as Singapore is an international travel hub with >6 million visitors annually.

CA-MRSA has only been isolated sporadically in Singapore, and no dominant clone was seen among our isolates. Singapore may be in an early phase of CA-MRSA emergence, and healthcare workers should remain vigilant for future outbreaks.
